# Learning from the covid-19 outbreaks in long-term care facilities: a systematic review

**DOI:** 10.1186/s12877-023-04319-w

**Published:** 2023-10-02

**Authors:** Helga Rafael Henriques, Diana Sousa, José Faria, Joana Pinto, Andreia Costa, Maria Adriana Henriques, Maria Cândida Durão

**Affiliations:** 1 Escola Superior de Enfermagem de Lisboa, CIDNUR – Nursing Research, Innovation and Development Centre of Lisbon, Avenida Prof Egas Moniz, 1600–190 Lisbon, Portugal; 2 Instituto de Saúde Ambiental – ISAMB, Lisbon Medical School - Avenida Professor Egas Moniz MB, 1649-028 Lisbon, Portugal

**Keywords:** COVID-19, Nursing, Homes for the aged, Infection control, Disease transmission, Infectious

## Abstract

**Background:**

The COVID-19 pandemic has devastatingly affected Long-Term Care Facilities (LTCF), exposing aging people, staff members, and visitors. The world has learned through the pandemic and lessons can be taken to adopt effective measures to deal with COVID-19 outbreaks in LTCF. We aimed to systematically review the available evidence on the effect of measures to minimize the risk of transmission of COVID-19 in LTCs during outbreaks since 2021.

**Methods:**

The search method was guided by the preferred reporting items for systematic reviews (PRISMA) and the reporting guideline synthesis without meta-analysis (SWiM) in systematic reviews. The search was performed in April 2023. Observational and interventional studies from the databases of PubMed, Web of Science, Scopus, Cochrane Systematic Reviews, CINAHL, and Academic Search were systematically reviewed. We included studies conducted in the LTCF with outbreaks that quantitatively assess the effect of non-pharmacological measures on cases of COVID-19. Two review authors independently reviewed titles for inclusion, extracted data, and undertook the risk of bias according to pre-specified criteria. The quality of studies was analyzed using the Joanna Briggs Institute Critical Appraisal.

**Results:**

Thirteen studies were included, with 8442 LTCF experiencing COVID-19 outbreaks and 598 thousand participants (residents and staff members). Prevention and control of COVID-19 infection interventions were grouped into three themes: strategic, tactical, and operational measures. The strategic measures reveal the importance of COVID-19 prevention and control as LTCF structural characteristics, namely the LTCF size, new admissions, infection control surveillance, and architectural structure. At the tactical level, the lack of personal and long staff shifts is related to COVID-19's spread. Operational measures with a favorable effect on preventing COVID-19 transmission are sufficient. Personal protective equipment stock, correct mask use, signaling, social distancing, and resident cohorting.

**Conclusions:**

Operational, tactical, and strategic approaches may have a favorable effect on preventing the spread of COVID-19 in LTCFs experiencing outbreaks. Given the heterogeneous nature of the measures, performing a meta-analysis was not possible. Future research should use more robust study designs to explore similar infection control measures in LTCFs during endemic situations with comparable outbreaks.

**Trial registration:**

The protocol of this systematic review was registered in PROSPERO (CRD42020214566).

**Supplementary Information:**

The online version contains supplementary material available at 10.1186/s12877-023-04319-w.

## Background

Long-term care facilities (LTCF) are characterized by being vulnerable to outbreaks of respiratory diseases, such as those caused by the influenza virus or the human coronavirus [1–3]. These institutions are frequently residences for older people who are socially and medically vulnerable to COVID-19 complications because of the interaction between advanced age and multimorbidity [4]. Additionally, LTCF-specific institutional and environmental characteristics, such as high occupancy density, shared living areas, people with cognitive and behavioral issues, a lack of human and material resources, and antiquated infrastructure, increase the sensitivity to COVID-19 [4]. LTCF includes nursing homes, skilled nursing facilities, retirement homes, assisted-living facilities, and residential care homes. In the European Union/European Economic Area (EU/EEA), before December 2019, there were 2.9 million residents in 43,000 of these LTCF types, representing 0.7% of the overall population [5].

The pandemic caused by SARS-CoV2 has devastatingly affected LTCFs, exposing aging people, staff members, and visitors [6–8]. More than 800,000 fatal cases of COVID-19 have been reported in EU/EEA LTCF since the beginning of the epidemic, with more than 88% affecting adults over 65 [9].

Despite high vaccination coverage, COVID-19 outbreaks continue to occur in LTCFs, including severe cases and fatalities. The vaccine is highly effective in preventing severe disease and death. However, current outbreaks in LTCF highlight the importance of early detection, rapid containment of COVID-19 outbreaks, and ensuring strict infection prevention and control measures [10, 11]. COVID-19 outbreaks in LTCF are defined as two or more cases linked by location and time, demonstrating an everyday exposure outside of a household [12].

Prominent international organizations have issued guidelines that guide these facilities to respond to the pandemic emergency [13, 14]. Still, these recommendations have not been subjected to the rigorous process of developing formal guidelines [15]. In the same way, the available systematic reviews on infection prevention and control measures in LTCF were mainly in 2020. They had a low level of evidence due to the design and quality of available studies [15–20]. Early studies were primarily focused on rapidly responding to the crisis rather than using interventional or experimental designs. Most of this research relied on case reports or cross-sectional methods and did not quantify the effect of containment measures in LTCF outbreaks. A recently published systematic review established the correlation between control measures and SARS-CoV-2 infection rates in residents and staff [21]. Although it sheds light on the subject, this review's limitations include a restricted time frame until November 2021, which precluded the inclusion of infection control measures adapted to the LTCF outbreak caused by new coronavirus variants, such as Omicron. Also, the databases used could lead to incomplete coverage of published studies. Therefore, it is not fully understood what measures prevent COVID-19 from spreading during COVID-19 outbreaks in LTCF. Some studies have already shown the need for an organized response to outbreaks in LTCF, concentrating attention on specific moments [22–24].

So, it is interesting to reflect on the answers to the following question: What measures favorably reduce COVID-19 transmission during outbreaks in LTCF?

The answers to this question may fill the current frameworks for decision-making [25, 26], enabling more scientifically supported future pandemic outbreak management practices.

We aim to systematically review the available evidence on the effect of measures to minimize the risk of transmission of COVID-19 in LTCs during outbreaks since 2021 We decided to concentrate on studies conducted since 2021 because we seek to distance ourselves from studies primarily focused on crisis response and lacking interventional or experimental designs [15–20]. Additionally, this timeframe allows us to account for the evolving understanding of the virus and its variants, including the emergence of the Omicron variant.

## Methods

This study has been undertaken as a systematic literature review based on the Preferred Reporting Items for Systematic Reviews and Meta-Analysis (PRISMA) [27] and the reporting guideline synthesis without meta-analysis (SWiM) in systematic reviews [28]. Its protocol was registered in the International Prospective Register of Systematic Reviews (PROSPERO) (CRD42020214566).

### Eligibility criteria

Our research question assumed a PICOT format (Population (P) /Intervention (I) /Comparison (C) /Outcome (O) / Time (T)): *which were the measures (I) implemented by LTCF with outbreaks (P) that* have a favorable effect on *reducing the risk of transmission of COVID-19 infection (O) since 2021 (T)? We understand that by* "Measures" any non-pharmacological structured intervention, norm, action, or procedure implemented in the LTCF to prevent and control the spread of COVID-19 during outbreaks, different from usual care.

We focused on when the virus entered and remained in the LTCF. We considered LTCF with outbreaks in all settings dedicated to adults and aging people, including residents, staff members, and visitors (Table 1). An outbreak was defined as ≥ 2 residents with laboratory-confirmed COVID-19. We included studies that allowed comparison across different groups, such as interventional studies (randomized or before and after) and observational studies (cohort and case–control) centers pediatric In this sense, we decided to exclude cross-sectional studies because they do not allow us to establish the temporal relationship between disease occurrence and exposure [29]. It is particularly troubling because of the instability of COVID-19 exposure conditions over time in LTCF, especially considering the introduction of new virus variants and the increasing vaccine coverage.

**Table 1:** Eligibility criteria

	**inclusion criteria**	**exclusion criteria**
Population	Studies conducted in the LTCF with outbreaks involving adults or aging residents, staff members, and their visits	Studies with the general population Studies conducted in hospitals, prisons, schools, community centers, primary care, or home-based care Pediatric residents (< 18 years)
Intervention	Non-pharmacological measures implemented in LTCF to safely minimize the risk of transmission of COVID-19 during outbreaks. Structured and planned care, different from usual care	The “measure” is not clear Pharmacological measures Other viral acute diseases with epidemic/pandemic potential include SARS, Middle East respiratory syndrome (MERS), and (pandemic) influenza
Outcome	Studies that quantitatively assess the effect of measures on the following outcomes: -Residents, staff members, or visitors SARS CoV2 + -Mortality related to SARS CoV2 -Hospitalization related to SARS CoV2 +	Other outcomes
Time	2021, 2022, 2023	Before 2021
Study design	Interventional studies (RCT or before and after studies) Observational studies (cohort and case–control)	Editorials, commentary, opinions, reviews, and book chapters Non-comparative studies (case reports or case series, qualitative studies) Cross-sectional studies Conference abstracts and summary reports Mathematical modeling studies
Language	English, French, Portuguese, and Spanish	Other languages

We decided to attend to Kesmodel's conclusion [29] "when cross-sectional data is used for analytical purposes, authors and readers should be careful not to make causal inferences, unless the exposure may safely be assumed to be stable over time" (p.388).

We excluded studies assessing other viral acute diseases, such as SARS, Middle East respiratory syndrome (MERS), and pandemic influenza. The most recent evidence has shown that, although there is epidemic/pandemic potential in these diseases, there are differences in pathogenicity that justify specific measures for preventing COVID-19 dissemination [16–18, 30, 31].

### Information sources and search strategy

We performed a literature search using the online databases of PubMed, Web of Science, Scopus, Cochrane Systematic Reviews, Cumulative Index to Nursing and Allied Health Literature (CINAHL), and Academic Search Complete. Previous reviews were consulted to create the current strategy [15–20]. The following medical subject headings (MeSH) and non-MeSH keywords were used as keywords in our search strategy, according to the PICOT domains, and using Boolean operators (Table 2). We searched in “ALL fields” to ensure better coverage. Because we wanted structured and planned care, different from usual care, we opted for using focused keywords on interventions. The search was performed on 2/04/2023 by one researcher (HRH).

**Table 2:** Search strategy

**Population**	(“Residential Facilities” OR “Assisted Living Facilities” OR “Homes for the Aged” OR “Nursing Homes” OR “Long-Term Care” OR “residential aged care” OR “Long-Term Care Facilities”)
Intervention	((“organization and administration” OR “health facility administration” OR “Guidelines” OR “practice guidelines” OR “Guidance” OR “Containment strategy” OR “Containment of Biohazards” OR “managed care programs” OR “training programs” OR “Risk assessment” OR "Guidelines as Topic" OR "Health Planning Guidelines" OR "Practice Guidelines as Topic" OR "Guideline") AND (“Disease Transmission” OR “Disease Transmission, Infectious” OR “Infectious Disease Transmission, Professional-to-Patient” OR “Infectious Disease Transmission, Patient-to-Professional” OR “Disease Transmission, Patient-to-Professional” OR “Disease Transmission, Professional-to- Patient” OR “Infection Control” OR “Risk Factors” OR “Risk reduction behaviour” OR “Harm reduction”)) AND (“COVID-19” OR “2019-nCoV” OR “SARS-CoV2”)
Comparison	No specific comparator was employed
Outcomes	(Patients OR Mortality OR morbidity OR “disease outbreak*” OR “cause of death” OR cases* OR hospitaliz* OR “attack rate^*1*^*” OR “case index”)*
Time	≥ 2021

1 We understand "attack rate" as a measure used in epidemiology to describe the frequency of new cases of a specific disease or condition within a population over a specified period. It is expressed as a percentage, representing the proportion of at-risk individuals developing the disease during the outbreak

In addition, we manually searched for potential records in the bibliographic reference list of systematic reviews on the topic in the previous search.

### Selection process

All detected references (identification) were exported to the Rayyan QCRI tool (Rayyan Systems Inc., Cambridge, MA, USA). It supported collaborative work throughout the team [32]. The research team was divided into small groups of two or three members who worked collaboratively on the screening process (AC, AH, CD, DS, HRH, JF, JP). The small teams' work was supervised by one researcher (HRH).

We started by removing duplicates. Then, two researchers (all team members) independently reviewed the title and abstract of the retrieved studies and decided if they met the predetermined eligibility criteria. We followed a decision tree that started by evaluating the type of study. If these criteria were met, we would proceed to the evaluation of the population. Once these criteria were met, we assessed the intervention, the outcome, and finally, the date of the study.

All articles that successfully met the eligibility criteria were evaluated during the full-text review. When two screeners disagree on whether an article fulfils the eligibility criteria, they resolve this disagreement through discussion. If the two screeners cannot reach a consensus for a particular article, a third person (HRH) acts as an arbitrator to decide on the contested article. The librarian was involved in finding articles that were not fully available.

### Data collection process and data items

Two researchers independently collected the data using a form that has been developed and piloted. Two researchers (HRH, CD) independently developed the searchable database, informed by the research question and the Taxonomic classification of planning decisions in health care [25]. These two versions were agreed upon and presented to the rest of the team in a meeting. All the team could improve and clarify the document to avoid misunderstandings or later disagreements.

Data related to study identification, research country, aim, study design, study period, LTCF involved, participants, outcomes, context, and conclusion were extracted.

Regarding the intervention, we categorized prevention and infection control measures as strategic, tactical, and operational measures according to the Taxonomic classification of planning decisions in health care [25].

Three teams of two reviewers extracted data from the included studies, and a third author resolved disagreements between the teams. At this point, to foster greater researcher consensus, each small group took on studies that were distinct from the ones they had previously examined. A third reviewer (HRH) double-checked all the data in the tables. Whenever data were unavailable, we contacted the study's author, requesting to provide this data. Data collection forms were designed using Microsoft Excel spreadsheets.

### Study risk of bias assessment

Each study was assessed independently according to the Joanna Briggs Institute (JBI) Critical Appraisal Checklist tools (Moola et al., 2020) [33].

The risk of bias disagreements was mitigated through discussion, which included the participation of a third author to ensure the quality of the appraisal process when necessary.

### Effect measures

All effects estimates were reported as OR or RR with 95% confidence intervals. When research provided information on both the unadjusted and adjusted intervention effects, we utilized the adjusted effects with information on the variables for which the models had been adjusted.

### Data synthesis

Given the methodological and clinical heterogeneity of the studies, each study was subjected to a descriptive analysis according to the "Synthesis Without Meta-analysis" (SWiM) reporting guideline [28]. Studies were grouped according to study design.

A narrative approach was used to describe the evidence, referring to the level of evidence supporting the interventions for each outcome in each domain of interest.

## Results

A total of 4053 references were exported from selected databases to the Rayyan application. We removed 151 duplicate records and concluded the first screening stage with 3902 records. After analyzing their titles and abstracts, we excluded 3816 records because they did not meet the eligibility criteria. After analysing full-text, we included 12 references.

**Fig. 1:**
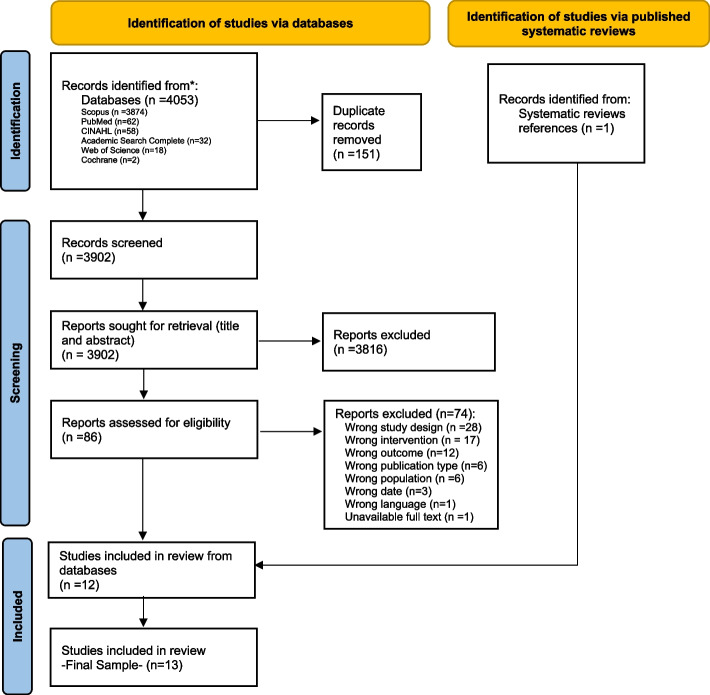
PRISMA Flowchart

In the excluded study sample, we identified six reviews on the topic [15–19,20 ]. Their lists of bibliographical references were manually analyzed, and from there, we included one extra-study (Fig. 1).

### Characteristics of included studies

After selection, thirteen studies were included, around 598 thousand participants (between residents and staff members) and 8442 LTCF, from the Asian (*n* = 2), American (*n* = 4), and European (*n* = 7) continents. One study reports multiple interventions. Eleven studies were observational, and two was interventional (Table 3).

**Table 3:** Characteristics of included studies

Characteristics	N (%)
Publication year:	
2021	10 (77.0)
2022	3 (23.0)
Location:	
Italy	3 (23.0)
England	3 (23.0)
United States of America	2 (15.0)
Canada	2 (15.0)
Germany	1 (8.0)
Iran	1 (8.0)
Japan	1 (8.0)
Design:	
Case–control study	5 (38.5.0)
Cohort study	6 (46.1)
Quase-experimental study	2 (15.4)
Prevention and infection control measures	
Single	12 (92.0)
Multiple	1 (8.0)

According to Joanna Briggs International's (JBI) criteria for critical appraisal, all articles had a low risk of bias. All studies fulfilled the JBI critical appraisal checklist, except for the control of some potential confounders.. Case–control studies responded to almost all JBI requirements [34–38]. Of these, only one of the manuscripts [37] clearly stated strategies for managing and controlling confounding factors. The cohort studies met most of the quality criteria outlined by JBI, demonstrating their alignment with established standards.. Still, we maintain reservations in two manuscripts [39, 40] about how confounding factors were stated, managed, and outcomes measured. Both manuscripts have evident gaps regarding how they used statistics to handle potential issues that could affect the results (confounding factors). Green et al. [39] lack explicit confounding factor variables, hindering intervention impact understanding. Zimmerman et al. [40] do not detail the methods used to measure exposure, the instruments employed, or the steps taken to ensure the accuracy and consistency of measurements.. One of the quasi-experimental studies [41] did not raise concerns; The second quasi-experimental study [42] exhibits limitations as it lacks consistent and comprehensive information regarding whether participants, in distinct comparison groups received comparable treatment or care, apart from the specified exposure or intervention being investigated. It is also unclear whether the follow-up was complete (Supplementary material).

We included all studies relevant to the research question that met the eligibility criteria (Table 4).

**Table 4:** Included studies

**ID Study author, year**	**Research Country**	**Study design**	**Aim**	**Study period**	**LTCF (n)**	**Participants**	**Outcomes**			Context	**Conclusions**
							**Covid + (n)**	**Deaths (n)**	**Hospitalized (n)**		
Vijh et al., 2021 [41]	Canada	Quasi-experimental study	To evaluate the effectiveness of a multisectoral intervention (a bundle of outbreak control measures) 14 days after implementation in terms of reducing subsequent transmission among residents and staff	February 28, 2020, and May 30, 2020	7	7 LTCF **Pré- intervention**: early outbreak period (RR, 1.07; 95% CI, 1.03–1.11; *p* < 0.001) **Post- intervention**: during the post-intervention period (RR, 0.73; 95% CI, 0.67–0.80; *p* < 0.001)	In total, 275 COVID-19 cases (165 staff and 110 residents) were reported to public health **Pré:** After adjusting for case type, there was a significant upward trend in the COVID-19 incidence rate during the early outbreak period (RR, 1.07; 95% CI, 1.03–1.11; p < 0.001) **Post:** A significant reversal in trend was identified (RR, 0.68; 95% CI, 0.62–0.75; p < 0.001). In particular, the postintervention trend demonstrated a 27% decrease in the COVID-19 incidence rate every 2 days (RR,0.73; 95% CI, 0.67–0.80; *p* < 0.001)	NR	NR	In British Columbia, 59% of COVID-19– related deaths were in LTCFs, compared to 75% in Canada overall and 30%–60% across Europe	Our study provides evidence for the effectiveness of this intervention to reduce the transmission of COVID-19 in LTCFs. This intervention can be adapted and utilized by other jurisdictions to protect the vulnerable individuals in LTCFs
Aghili et al. 2022 [34]	Iran	Case–control study	To identify the predictors of contracting COVID-19 among older people in nursing homes in Iran	From 25 March to 12 July 2021	6	409 residents **Cases**: 136 residents Covid + **Control**: 273 residents Covid-	**Cases**: 136 **Control**:273 covid-	NR	NR	There are no formal statistics about the mortality rate due to COVID-19 in nursing homes in Iran. Nursing homes are predominantly non-government-owned organizations that are authorized, supervised and funded by the State Welfare Organization of Iran	The results indicate that an increase in older people becoming infected by SARS-CoV-2 in nursing homes is probably because of not wearing a mask in common places, use of a cloth mask, longer staff shift durations and not having a glass shield when interacting with visitors from outside of nursing homes
Telford et al., 2021 [35]	USA	Case–control study	Determine the efficacy of recommendations for infection prevention and control of COVID-19 in LTCF for reducing transmission among high-risk populations	June and July 2020	24	24 LTCF **Cases: **11 LTCF, 1310 residents Higher-prevalence group (HPG) (infection prevalence among residents greater than the overall infection proportion (39%)) **Control: **13 LTCF, 1270 residents—Lower-prevalence group (LPG) (infection prevalence lower than the overall proportion)	**Cases**: HPG – 817 **Control**: LPG – 187	**Cases**:HPG124 **Control**: LPG – 38	**Cases**: HPG – 196 **Control**: LPG – 51	Fulton County, Georgia, which covers the city of Atlanta, had received reports of 1,183 COVID-19 infections among residents from 45 LTCFs within its jurisdiction; 51% of COVID-19 deaths in Fulton County were attributed to LTCF residents	LTCFs with lower COVID-19 prevalence among residents had a significantly greater implementation of IPC recommendations compared to those with higher COVID-19 prevalence
Wang et al., 2021 [36]	USA	Case–control study	This study compares key nursing home characteristics, infection prevention and control deficiencies, and five-star ratings among Florida nursing homes with and without resident COVID-19 cases and deaths. The study further examines the association between facility and resident characteristics, quality indicators, and COVID-19 cases and deaths	July to October 2020	686	686 Nursing Homes (2 reports: A—July 26, 2020; B- October 25, 2020) **Cases1: 499 **Nursing Homes with at least one confirmed resident COVID-19 case **Control1: 187 **Nursing Homes with no COVID-19 cases **Cases2: 271**Nursing Homes with at least one resident COVID-19 death **Control2: 415** Nursing Homes with no COVID-19 deaths	**Cases**: A-499* B-629* **Control**: A-187 B-57 *COVID-19 cases was coded as “yes” if the nursing home had at least one confirmed resident COVID-19 case	**Cases**: A-271** B-456** **Control**: A-415 B-271 **COVID-19 deaths was coded as “yes” if at least one resident COVID-19 death was reported in the nursing home	NR	Approximately one-fifth to two-fifths of all COVID-19 deaths in the U.S. occur in nursing homes or other long-term care facilities	The likelihood of having COVID-19 cases is related to facility size, average occupancy rate, infection prevention and control deficiencies and low nurse staffing hours per resident day
Ohta et al. 2021 [37]	Japan	Case–control study	To clarify the effect of coronavirus disease (hereafter, COVID-19) control on patients’ health conditions and staff’s working conditions in rural nursing homes	1 April 2019 to 31 March 202	1	96 participants: all the staff and patients in a rural nursing home **Cases: **48 pre-COVID-19 group (2019–2020) **Control: ** 48 post-COVID-19 group (2020–2021)	NR	NR	**Visits to the outpatient department** **Cases** (Post-COVID (2020–2021): 210 visits **Control** (Pre-COVID (2019–2020): 261 visits **Visits to the emergency department** **Cases** (Post-COVID (2020–2021):62 visits **Control **(Pre-COVID (2019–2020) :66 visits	During the study period, 287 persons were infected with COVID-19 in Shimane prefecture	This study showed that the implementation of strict infection control measures against COVID-19 in a rural nursing home can reduce the contact between the nursing home patients and healthcare staff, without an increase in the number of patients experiencing emergency situations. Due to the low statistical power of this study, we did not observe any statistically significant results
Orlando 2022 [38]	Italy	Case–control	To understand which organizational –structural characteristics of nursing homes and the preventative measures adopted in response to the pandemic are associated with the risk of a COVID-19 outbreak	March–December 2020	100	**Cases**: 20 LTCF reported an outbreak COVID-19 **Control**: 80 LTCF without COVID-19	**Cases:** 20 LTCF reported an outbreak COVID-19 **Control:** 80 LTCF without COVID-19	NR	NR	The article does not provide any local or national statistics	There was evidence of a higher risk of COVID-19 in larger facilities and when new patients were admitted during the pandemic. All other structural–organisational characteristics and preventative measures were not associated with an outbreak
Stemler et al., 2022 [42]	Germany	Quasi-experimental study	To investigated whether repeated non-mandatory RT-PCR SARS-CoV-2 surveillance of Nursing Homes (NH) staff and visitors reduces COVID-19 incidence rates in NH residents and allows to reduce visiting restriction	From early October 2020 to mid-December 2020 at maximum	4 (2 Interventional Nursing Homes (INH); 2 control Nursing Homes (CNH))	**Cases:** 2 INH 260 residents 335 Health Care Workers (HCW) (865 tests) 3150 visitors (722 tests) **Control**: 2 CNH 261 residents 425 HCW 3649 visitors	**Cases**: 2 INH Staff – 23 Visitors – 3 **Control**: 2CNH NR	**Cases**: Residents –23 (8.8%) **Control**: Residents – 4 (1.5%)	NR	At the beginning of the study period, the local incidence of SARS-CoV-2 in the City of Cologne was 99.4 infections/100,000 inhabitants/week, then rose to a maximum of 227.9 infections/100,000 inhabitants/week on October 30th, subsequently decreased to a lowest level of 129.3 infections/100,000 inhabitants/week on November 27th, and then rose again to 161.8 infections/100.000 inhabitants/week by December 18th, 2020	In a real-world setting without available rapid testing, voluntary RT-PCR SARS-CoV-2 testing of HCW and visitors does not prevent COVID-19 outbreaks in NH. Complete, non-selective testing for these groups should be instituted before visiting restrictions can be reduced
Shallcross et al., 2021 [46]	England	Cohort study	To identify factors associated with SARS-CoV-2 infection and outbreaks among staff and residents in LTCFs	Between May 26 and June 19, 2020	5126	Residents-160033 Staff-248594	Residents-19571 Staff-10630 LTCF at least with one COVID-19 case-2724 Large outbreack-469	NR	NR	LTCFs, which provide care to the elderly and those with disabilities have experienced among the highest rates of SARS-CoV-2 infection, and account for 30–50% of all COVID-19 related deaths in countries including the USA, England, Scotland, France, Spain, and Sweden In the UK, there are an estimated 400,000 residents living in approximately 11,000 LTCFs for the elderly	Reduced transmission from staff is associated with adequate sick pay, minimal use of agency staff, an increased staff-to-bed ratio, and staff cohorting with either infected or uninfected residents. Increased transmission from residents is associated with an increased number of new admissions to the facility and poor compliance with isolation procedures
Lombardo et al., 2021 [74]	Italy	Cohort study	To collect information on the spreading and impact of severe acute respiratory syndrome coronavirus 2 (SARS‐CoV‐2) infection in nursing homes, and on how suspected and/or confirmed cases were managed	From March 25 to May 5, 2020	1356	100,806 residents	LTCF: 161 had residents with SARS‐CoV‐2 positive; 381 had had at least one resident with flu‐like symptoms; 278 had SARS‐CoV‐2 positive staff members	Residents: 680 COVID + 3092 flu‐like symptoms	Residents: 965 SARS‐CoV‐2 +; 2021 flu‐like symptoms	Italy reported 245,864 cases of COVID-19 and 35,102 related deaths	Larger facility size was significantly (*p* < 0.05) related to increased probability of having COVID‐19 cases Some critical issues characterized NHs with COVID‐19 outbreak compared to ones without an outbreak, such as lack of personnel, difficulty in transferring to the hospital or other facility patients or isolating them in a single and NHs with a higher number of beds
Green et al., 2021 [39]	England	Cohort study	To describe the epidemiology and transmission of COVID-19 in outbreak free care homes	April and May 2020	34	714 residents	22 residents	NR	NR	The article does not provide any local or national statistics	The use of agency staff was associated with an eightfold increased risk and this needs to be considered in future support to care homes. Closing shared spaces was not associated with an increased risk of infection
Cazzoletti et al., 2021 [44]	Italy	Cohort study	To examine the association between certain measurable factors (structural, organizational and practice-related) and the cumulative incidence of COVID-19 among nursing home residents in the Autonomous Province of Trento, Italy, during the peak of the COVID-19 outbreak, the so-called “first wave” (March–May 2020)	From 1 March 2020 to 1 June 2020	45	4158 residents	**Cases**: 37 LTCF with an outbreak **Control**: 20 LTCF without an outbreak	NR	NR	The number of long-term care beds per 100,000 people aged ≥ 65 is two times greater than the national average (38.5 in the Autonomous Province of Trento versus 14.6 in Italy) This study performed the evaluation in a timeframe covering the entire “first wave” of the COVID-19 epidemic, when containment measures were not fully in place. The cumulative incidence of COVID-19 was higher in the nursing home facilities located in the western area of the province, which borders the Lombardy region, the most affected area during the first phase of the pandemic	Structural/organizational factors and standard IPC measures may not predict the epidemiology of COVID-19 outbreaks and be sufficient alone to protect nursing homes against them
Zimmerman et al., 2021 [40]	USA	Cohort study	This cohort study compared rates of COVID-19 infections, COVID-19 admissions/readmissions, and COVID-19 mortality, among Green House/small nursing homes with rates in other nursing homes	Between January 20, 2020 and July 31, 2020	435	A-Green House/small Nursing Homes—43 B-Traditional Nursing Homes with < 50 beds—177 C-Traditional Nursing Homes with ≥ 50 beds—215	COVID-19 cases per 1000 resident days (90th Percentile): A = 0,30 B = 1,61 C = 2,10 A vs B – *p* = 0,014 A vs C *p* < 0,001	COVID-19 mortality per 100 positive cases (median): A = 0 B = 10 C = 12.5 A vs B – *p* < 0,001 A vs C *p* < 0,001	COVID-19 admissions/ readmissions per 1000 resident days (90th Percentile): A < 0.01 B = 0,74 C = 0,49 A vs B – *p* = 0,007 A vs C *p* = 0,007	As of January 8, 2021, 6% of COVID-19 cases, and 38% of COVID-19 deaths, have been attributed to long-term care	COVID-19 incidence and mortality rates are less in Green House/small nursing homes than rates in traditional nursing homes with < 50 and ≥ 50 beds, especially among the higher and extreme values. Green House/small nursing homes are a promising model of care as nursing homes are reinvented post-COVID
Brown et al., 2021 [45]	Canada	Cohort study	To develop a reproducible index of nursing home crowding and determine whether crowding was associated with COVID-19 cases and mortality in the first months of the COVID-19 epidemic	From March 29 to May 20, 2020	618 Low Crowding Index – 310 nursing homes Hight Crowding Index – 308 nursing homes	78 607 residents Low Crowding Index – 46,028 residents Hight Crowding Index – 32 579 residents	5218 (6.64%) residents Covid + Low Crowding Index – 2071 (4.50%) residents **Vs** Hight Crowding Index – 3147 (9.66%) residents (*p* < 0,001)	1452 (1.85%) residents died Low Crowding Index – 578 (1.26%) residents **Vs** Hight Crowding Index –874 (2.68%) residents (*p* < 0,001)	NR	Recent estimates suggest that nursing home residents comprise approximately 35% of COVID-19 deaths in the US and between 66 and 81% of deaths in Canada. Compared with community-dwelling older adults, nursing home residents are 5 times more likely to die of COVID-19	Shared bedrooms and bathrooms in nursing homes are associated with larger and deadlier COVID-19 outbreaks

*CI* Confidence Interval, *CNH* Control Nursing Homes, *HCW* Health Care Workers, *HPG* Higher-prevalence group, *IPC* Infection prevention and control, *INH* Interventional Nursing Homes, *LPG* Lower-prevalence group, *LTCF* Long-Term Care Facility, *NH* Nursing Homes, *NR* Not reported, *OD* Odds ratio, *p* – p value, *RR* Rate Ratio

The interventions for preventing and controlling COVID-19 infection were grouped into three themes, as proposed by the taxonomy of Hulshof et al. (2012), for decision-making about the planning and control of health resources: strategic measures, tactical measures, and operational measures. The category of strategic measures includes actions that concern the organizational management structure. Tactical measures refer to those applied to team management. Operational measures are related to the management of residents’ care. Given the significant variations in the type of interventions and their effects on LTCs during COVID-19 outbreaks, we have subcategorized the impact of these interventions: unfavorable effect, favorable effect, or null effect. This approach provides a comprehensive understanding of the direction of the effect of different interventions in controlling COVID-19 spread in LTCs during recent outbreaks(Table 5).

**Table 5:** Sub-group analyses of type of measures with unfavorable/favorable/null effects on COVID-19 transmission

ID Study author, year	Statistical analysis	Type of measures	Measures with an unfavorable effect	OR ( 95% CI)	Measures with a favorable effect	OR ( 95% CI)	Measures without significant effect
Vijh et al., 2021 [41]	A mixed-effect segmented Poisson regression was fitted to our facility-specific COVID-19 case data against time to assess the association between the intervention and the COVID-19 incidence rate. The model was built using a standard approach for segmented regression of time series data	**Strategic**					A series of outbreak measures are classified into four categories: case and contact management, proactive case detection, rigorous infection control practices, and resource prioritization and stewardship
		**Tactic**					
		**Operational**					
Aghili et al. 2022 [34]	Bivariate logistic regression models were performed amongst the variables of infection prevention and control principles as predictor variables, and get COVID-19 as the dependent variable (yes/no). A multivariate logistic regression model was used to identify independent predictors of getting COVID-19 using variables with p values less than.05. The final model was obtained after removing all non-statistically significant variables (enter the selection procedure)	**Strategic**					Number of beds in the room Meals places Surface disinfection
		**Tactic**	Longer staff shifts (vs. No)	3.02 (1.68–5.43), *p* < 0.001			Education for COVID-19
		**Operational**	Not using a mask outside the room (vs. Yes)	3.37 (1.74–6.53), *p* < 0.001	Glass in visitors’ space (vs. No)	1.95 (1.11– 3.50), *p* = 0.025	Flu vaccine, Flu history Using a mask inside the room Mask wearing method Mask change time (daily) Physical distance with no roommate Number of hand washing (daily) Hand washing time (seconds) Travel history in the last month, Take vitamin D
			Cloth mask or nothing (vs. Simple surgical mask)	2.47(1.13–5.42), *p* = 0.024			
Telford et al., 2021 [35]	Frequency distribution (counts and percentages) was used to describe the overall LTCF adherence to each key indicator. The implementation of key indicators within each category was also calculated as a composite proportion of all possible indicators that could be adhered to within the category A chi-squared test of proportions was used to test for differences between the higher and lower prevalence groups, with P.05 set as the cutoff for statistical significance A two-tailed t-test for two independent means was used to determine statistical differences between groups for continuous variables, with p.05 selected as the level of significance	**Strategic**			Bathroom and sink inside the bedroom (HPG 73% vs. LPG 100%, *p* = 0.04)		Disinfection
		**Tactic**			Training and frequent audits are conducted to ensure proper mask use by staff members (HPG 36% vs. LPG 85%, *p* = 0.02)		
					Staff is trained, and audits take place to ensure proper donning and doffing of PPE (HPG 55% vs. LPG 92%, *p* = 0.03)		
		**Operational**			Distancing from others (HPG 54% vs LPG 74%, *p* = 0.01)		Symptom Screening Hand Hygiene
					PPE category (HPG 41% vs. LPG 72%, *p* = 0.01)		
					Masks are used properly by staff inside the COVID unit (HPG 45% vs. LPG 100%,*p* < 0.01)		
Wang et al., 2021 [36]	Separate logistic regression models were built to estimate the association between infection prevention and control deficiencies, the overall five-star rating, the presence of COVID-19 resident cases, and, among nursing homes with one or more cases, the presence of an outbreak The multicollinearity of independent variables was examined using the variance inflation factor. Odds ratios (ORs), 95% confidence intervals (CIs), and significant levels were reported	**Strategic**	Higher average occupancy rate (vs. lower)	21.24 (1.08, 418.13)–31.19 (3.82, 254.54), *p* = 0.05–0.01, for COVID + cases	Facilities ≤ 60 beds (vs. > 121 beds)	0.13 (0.03, 0.52)– 0.20 (0.10, 0.40), *p*. < 0.001–0.1, for Covid + cases	
			Prevention and control efficiency > 1 citation (vs. ≤ 1 citation)	2.09 (0.95, 4.60, *p* = 0.068 for Covid + cases)	Facilities between 61–120 beds	0.27(0.08, 0.94) –0.53(0.32, 0.87), *p* < 0.05, for Covid + cases	
		**Tactic**	Low nurse staffing hours per resident per day (vs. higher)	0.67 (0.44, 1.04), *p* = 0.1 for the COVID + outbreak			
		**Operational**					
Ohta et al. 2021 [37]	The differences in participant characteristics, frequency of patients’ medical care visits to the outpatient and emergency departments, and the number of days off taken by the staff between the pre and post-COVID-19 control groups were analyzed using t-tests and chi-squared tests. CCI was categorized binomially (5 or > 5) to assess the severity of medical conditions. For all comparisons, the level of statistical significance was set at p 0.05	**Strategic**					Usage of information and communication technology. An ICT system called “Mame-net” was used to share patient information between the clinic and the nursing home
		**Tactic**					Daily Monitoring of the Staff’s Health Conditions The staff and clinic physicians monitored their fever and symptoms daily and note down their conditions on a checklist. The checklists were monitored, and if they had mild symptoms or fever > 37 ◦C, they were not permitted to work in the nursing home
		**Operational**					Contact Limitation To reduce the risk of infection transmission, care workers wore facemasks, plastic gloves, and face shields, and used hand sanitizers every time they cared for their patients The frequency of care was reduced from three times/day to two times/day. Regarding mealtimes, the patients usually ate their food in the lounges; however, they were now required to eat in their respective rooms during the pandemic. Further, the patients’ families were restricted from meeting the patients, except in emergency situations
Orlando 2022 [38]	A binary logistic regression model evaluated the association between potential risk factors with the probability of reporting at least two positive cases among residents	**Strategic**	> 15 beds (vs < 15 beds)	5.37 (1.58 to 22.8), *p* < 0.05, for COVID-19 outbreak			Presence of multiple rooms The proportion of single rooms Separate entrances Presence of isolation environment Active surveillance of staff Presence of written operational procedure for the management of cases External cleaning company Presence of a written operational procedure for new admissions Access by external suppliers Opened to visitors post-first lockdown
		**Tactic**					Frequency of shifts staff Presence of a grey area for healthcare and non-healthcare staff Dressing rooms for staff Trained staff on procedures to contain COVID-19 Trained residents on procedures to contain COVID-19
		**Operational**					Use of personal protective equipment Active surveillance for guests
Stemler et al., 2022 [42]	We evaluated the occurrence of symptomatic SARSCoV-2 incidence among residents in both INH and CNH as the primary endpoint (with an outbreak defined as the occurrence of ≥ 1 SARS-CoV-2-infected resident in a timely and situational context). Qualitative data were summarized by absolute and relative (%) frequency and quantitative data by a median and interquartile range (IQR) Differences in categorical frequency distributions were only tentatively evaluated using the Chi-square test since the assumption of independent observations is untenable, and more adequate methods require more data	**Strategic**					Regular and voluntary RT-PCR SARS-CoV-2 testing of HCWs and visitors
		**Tactic**					
		**Operational**					
Shallcross et al., 2021 [46]	A multivariable logistic regression model was performed to identify factors associated with infection in staff and residents	**Strategic**	Increase in the number of new admissions (vs baseline)	Infection in residents (1·01 [1·01–1·01], *p* < 0·0001) and staff (1·00 [1·00–1·01], *p* = 0·0005), and of outbreaks (1·08 [1·05–1·10], *p* < 0·0001)			
		**Tactic**	Staff often or always cared for both infected or uninfected residents (vs. cohorted staff with either infected or uninfected residents)	The odds of infection in residents (1·30 [1·23–1·37], p < 0·0001) and staff (1·20 [1·13–1·29], *p* < 0·0001), and of outbreaks (2·56 [1·94–3·49], *p* < 0·0001) were significantly higher	Paid staff statutory sick pay (vs No)	The odds of infection in residents 0·80 [ 0·75–0·86], *p* < 0·0001), staff 0·70 [0·65–0·77], *p* < 0·0001), Large outbreaks 0·59 [0·38–0·93], *p* = 0·024) were significantly lower	
			Frequent employment of agency nurses or carers (vs no employment of agency nurses or carers)	Significantly increased odds of infection in residents (1·65 [1·56–1·74], *p* < 0·0001) and staff (1·85 [1·72–1·98], *p* < 0·0001), and of outbreaks (2·33 [1·72–3·16], *p* < 0·0001) and large outbreaks (2·42 [1·67–3·51], *p* < 0·0001)	Increase in the staff-to-bed ratio (vs No)	Reduced odds of infection in residents (0·82 [0·78–0·87], *p* < 0·0001) and staff (0·63 [0·59–0·68], *p* < 0·0001	
		**Operational**	Difficulties in isolating residents (vs. No)	Significantly higher odds of infection in residents (1·33 [1·28–1·38], p < 0·0001) and staff (1·48 [1·41–1·56], p < 0·0001), and of outbreaks (1·84 [1·48–2·30], *p* < 0·0001) and large outbreaks (1·62 [1·24–2·11], p = 0·0004)			
Lombardo et a., 2021 [43]	An univariate and a multivariate regression logistic model were performed to assess whether critical aspects and characteristics of the NHS, adjusted for a geographical area, were associated to COVID‐19 outbreaks defined as the presence of laboratory‐confirmed cases among deceased and hospitalized residents or staff members, and among residents currently living in the facility	**Strategic**					Lack of laboratory tests Lack of PPE
			Median number of beds > 60 (vs. < 60 beds)	1.50 (1.09–2.07), *p* = 0.013			
		**Tactic**	Lack of personnel (vs. No)	3.22 (2.38–4.36), *p* < 0.001			
		**Operational**	Difficulty in isolating (vs. N)	1.97 (142–2.73), *p* < 0.001			
			Difficulty in transferring (vs. N)	4.66 (2.98–7.31), *p* < 0.001			
Green et al., 2021 [39]	Where the prevalence of positive residents was high enough, a Poisson regression model was created to explore the above variables while accounting for care home differences Where the prevalence was too low to allow appropriate stratification, univariable analysis was undertaken using Fisher’s exact test dependent on the numbers within the contingency tables	**Strategic**					Closing residents shared space
		**Tactic**	Employing agency staff was more likely to contain test-positive residents	8.4 (1.2–60.8)			
		**Operational**					
Cazzoletti et al., 2021 [44]	The association between median cumulative incidence of COVID-19 cases among residents and characteristics of nursing homes was assessed by Mann–Whitney U test, Kruskal–Wallis test or Spearman rho. To evaluate the potential confounding of geographical area, a 2-level random intercept logistic model was fitted, with level 1 units (patients in nursing homes) nested into level 2 units (nursing homes), and “being a COVID-19 case” as the dependent variable	**Strategic**			The nursing homes with no cases of COVID-19 were those that were more likely to implement outbreak management procedures compared to homes with at least 1 case of COVID-19	Nursing homes with implement outbreak management procedures (23.5%), vs nursing homes with at least 1 case of COVID-19 (3.6%), p = 0.060	Facility size Single-occupancy rooms Policies for the management of personnel at risk of infection Official protocols/procedures on Infection control and prevention Established an infection surveillance program Procedure on standard and additional precautions
		**Tactic**					Full-time equivalent nurses, physicians, aid staff Training of staff on the management of occupational exposures to biohazards Training of staff on the correct hand hygiene procedure Training of staff on how to prevent the spread of respiratory infections Training of staff on the correct use of PPE
		**Operational**					Conformity to quality standards Compliance with operations of routine and terminal cleaning/sanitation/disinfection Availability of hand hygiene supplies Regular checks of the quality of the cleaning/sanitation/disinfection Hand hygiene Use of PPE Isolation measures Sanitation Procedure for management of residents with suspected communicable diseases
Zimmerman et al., 2021 [40]	For each COVID-19 outcome (cases, admission/readmission, mortality), a log-rank test, which is a nonparametric test that emphasizes detection of group differences among higher values, was applied to compare rates among the 3 Nursing Home types. Multiple comparisons between 2 groups (Green House/small Nursing Homes vs traditional Nursing Homes < 50 beds; Green House/small Nursing Homes vs traditional Nursing Homes ≥ 50 beds) were subsequently performed using log-rank tests if the omnibus test was statistically significant at *p* < 0,05. Statistical significance for the pairwise comparisons was set at *p* < 0,025, per Bonferroni adjustment	**Strategic**			Non-traditional small house Nursing Homes model: 10 to 12 residents and have consistent and universal staff assignment (thereby limiting ancillary staff), private rooms and bathrooms, smaller overall space, and a central entry	COVID-19 cases are lower in Non-traditional small house Nursing Homes (Compared with < 50 Beds – *p* = 0,014; Compared with ≥ 50 Beds, *p* < 0,001) COVID-19 admissions/ readmissions are lower in Non-traditional small house Nursing Homes (Compared with < 50 Beds – *p* = 0,007; Compared with ≥ 50 Beds, *p* = 0,007) COVID-19 mortality are lower in Non-traditional small house Nursing Homes (Compared with < 50 Beds – *p* < 0,001; Compared with ≥ 50 Beds, *p* < 0,001)	
		**Tactic**					
		**Operational**					
Brown et al., 2021 [45]	All analyses were conducted using SAS, version 9.4 (SAS Institute), and all reported P values were based on 2-sided testing. P values less than .05 were considered statistically significant. A quasi-Poisson regression was used to model cases and deaths using the logarithm of the number of beds in the home as an offset, while logistic regression was used to model introduction of COVID-19 into the home The nursing home crowding index was defined as the mean number of occupants per room and bathroom across an entire home according to the following equation: Nresidents ÷ (½Nbedrooms + ½Nbathrooms). This translated to weights per resident according to the room they occupied: single-occupancy room with private bathroom (1); single-occupancy room with a shared bathroom (1.5); double-occupancy room (with shared bathroom) (2); and quadruple-occupancy room (4)	**Strategic**	Hight Crowding Index [double-occupancy room (with shared bathroom); or quadruple-occupancy room]	Compared with a home with low Crowding index, homes with hight Crowding Index had the double of the COVID-19 incidence (relative risk [RR], 2.05; 95% CI, 1.49–2.70) and is associated with COVID-19 mortality (RR, 1.97; 95% CI, 1.36–2.84)			
		**Tactic**					
		**Operational**					

*CI* Confidence interval, *HPG* Higher-prevalence group, *LPG* Lower-prevalence group, *LTCF* Long-term care facilities, *OR* Odds ratio, *p* p value, *RR* Rates ratio

Since the set of studies reported the implementation of very different measures, the data collection time of each study varied greatly, and the participants probably had different vaccination coverage, we considered the sample extremely heterogeneous. Therefore, a synthesis without meta-analysis was performed [28].

### Strategic measures

There appears to be an indication that facility size might significantly predict COVID-19 in LTCF [36, 38, 40, 43]. However, further analysis and investigation are needed to confirm this finding definitively [36, 38, 40, 43, 44].

LTCF with no more than 60 beds (OR = 0.13–0.20) and 61–120 beds (OR = 0.27–0.53) had lower COVID-19 cases than those with 121 or more beds [36]. Lombard et al. [43] also concluded that LTCF with a median number of beds > 60 (vs. < 60 beds) had an odd infection of 1.50 (1.09–2.07), *p* = 0.013. Moreover, Orlando et al. [38] found that the risk of reporting an outbreak was 5.37 times greater (1.58 to 22.8) in facilities with more than 15 beds than in those with less than 15 beds. Additionally, COVID-19 cases are lower in LTCF with 10–12 residents (vs. < 50 Beds – *p* = 0,014; ≥ 50 Beds, *p* < 0,001), as well as COVID-19 hospital admissions/ readmissions (vs. < 50 Beds – *p* = 0,007; ≥ 50 Beds, *p* = 0,007) or COVID-19 mortality (vs. < 50 Beds – *p* < 0,001; ≥ 50 Beds, *p* < 0,001) [40].

The studies do not agree on the importance of the number of beds per room. Some results show that the number of beds per room has no significant association with the COVID-19 spread [34, 38, 39, 44]. However, others show that double or quadruple-occupancy rooms (with shared bathrooms) promote COVID-19 dissemination (compared with LTCF with single rooms, had double the COVID-19 incidence (relative risk [RR], 2.05; 1.49–2.70) and is associated with COVID-19 mortality (RR, 1.97; 1.36–2.84)) [45].

Higher average occupancy rates were associated with increased COVID-19 cases (OR = 21.24–31.19) [36]. Maximum occupancy limits in small, enclosed spaces, such as elevators, dressing/dining rooms, and WCs inside the bedroom, were associated with a lower prevalence of COVID-19 infection in LTCF with an active outbreak [35].

The data suggest a potential association between new admissions and increased odds of infection [46], highlighting the importance of testing and isolating residents upon entry to the LTCF.. Outbreak testing could prevent 54% (weekly testing with a 48-h turnaround) to 92% (daily testing with immediate results and 50% relative sensitivity) of SARS-CoV-2 infections.

Regular and voluntary RT-PCR SARS-CoV-2 testing of healthcare workers and visitors seems to have no significant effect on COVID-19 prevention in LTCF [42].

Facilities with multiple Infection prevention and control (IPC) deficiencies were more likely to report COVID-19 cases (OR:2.09 [0.95, 4.60], *p* = 0.068) than those with only one IPC deficiency [36]. Similarly, LTCFs with no cases of COVID-19 were those who were more likely to implement outbreak management procedures compared to homes with at least 1 case of COVID-19 (*p* = 0.060) [44].

Surface disinfection [34, 35], closing residents' shared spaces [39], and meal places [34] seems to have no significance in the prevention of COVID-19 infection, as open to visitors post-first lockdown [38] and the use of information and communication technology to share patient information between the clinic and nursing home [37]. Likewise, official protocols/procedures on infection control and prevention, policies for managing personnel at risk of infection, an infection surveillance program, or procedures on standard and additional precautions appear to have no significant impact on the management of the outbreak [44].

The data suggest that a multicomponent intervention, like case and contact management, proactive case detection, rigorous infection control practices, and resource prioritization and stewardship, led to a reduction in the transmission of COVID-19 in LTCFs [41].

### Tactical measures

Longer staff shifts seem to be a predictor of getting COVID-19. The infection rate was almost three times greater in nursing homes with longer staff shifts than in those that did not (OR 3.02 (1.68–5.43), *p* < 0.001) [34]. The odds of infection in residents (1.30 [1.23–1.37], *p* < 0.0001) and staff (1.20 [1.13–1.29], *p* < 0.0001), as well as outbreaks (2.56 [1.94–3.49], *p* < 0.0001), were significantly higher in LTCF where staff frequently or always cared for both infected and uninfected residents, compared to those where staff cohort with either infected or uninfected residents [46].

Total nurse staffing hours per resident per day were found to be higher in nursing homes reporting no COVID-19 outbreak (OR 0.67 (0.44, 1.04), *p*. = 0.1) [36]. An increase in the staff-to-bed ratio was associated with reduced odds of infection in residents (0.82 [0.78–0.87], *p* < 0·0001) and staff (0.63 [0.59–0.68], *p* < 0·0001) [46].

In LTCFs that provided staff statutory sick pay compared to those that did not, the risks of SARS-CoV-2 infection were significantly lower in residents (adjusted odds ratio [aOR] 0.80 [0.75–0.86], *p* < 0.0001), staff (0.70 [0.65–0.77], p00001), and large outbreaks (0.59 [0.38-0.093], *p* = 0.024) [46].

The lack of personnel was associated with COVID-19 infection (OR = 3.22 [, 2.38–4.36], *p* < 0.001) [43]. Also, employing agency nurses or caregivers frequently was associated with significantly increased odds of infection in residents (OR 1.65 [1.56–1.74], *p* < 0.0001) and staff (1.85 [1.72–1.98], *p* < 0.0001), and of outbreaks (2.33 [1.72–3.16], *p* < 0.0001) and large outbreaks (2.42 [1.67–3.51], *p* < 0.0001), compared with no employment of agency nurses or carers [46]. In agreement, the study by Green et al. [39] concluded that LTCF-employing agency staff was at a greater risk of having residents test positive (RR 8.40, 1.16–60.84). However, full-time nurses, doctors, or aid staff have no association with the number of confirmed cases of covid-19 in LTCF [44].

Training staff on managing occupational exposures to biohazards, the correct hand hygiene procedure, how to prevent the spread of respiratory infections, and using personal protective equipment had no association with the median cumulative incidence of COVID-19 cases among residents [44]. Nevertheless, training, and frequent audits for proper donning/doffing of PPE (*p*. = 0.03) and mask use (*p*. = 0.02) occurred more in lower-prevalence COVID-19 infection LTCFs [35]. Staff members in 100% of lower-prevalence LTCFs were observed to use masks properly in the COVID-19 unit compared to 45% in the higher-prevalence group (*p* < 0.01) [35]. However, training staff and residents on procedures to contain COVID-19 [38] and daily monitoring and reporting of the staff's health conditions (fever and symptoms) [37] seems to have no significant effect on COVID-19 prevention.

### Operational measures

Isolating residents appears to be a critical component of COVID-19 prevention in LTCF. Compared with LTCF that did not report difficulties in isolating residents, those that did had significantly higher odds of infection in residents (1.33 [1.28–1.38], *p* < 0.0001), staff (1.48 [1.41–1.56], *p* < 0.0001), outbreaks (1.84 [1.48–2.30], *p* < 0.0001), and large outbreaks (1.62 [1.24–2.11], *p* = 0.0004) [46]. Also, the conclusions of the study by Lombardo et al. [43] indicate that difficulties in isolating residents (OR:1.98, *p* < 0.001) are associated with no COVID‐19 infection. Social distancing had a significantly higher implementation (*p*. < 0.01) in the lower COVID-19 prevalence LTCF [35].

However, the favorable and significant effect of resident isolation has not been consistently demonstrated [37]. Based on these conclusions, the package measures aimed at reducing the risk of infection transmission (which included care workers using personal protective equipment and reducing the frequency of care from three times per day to two times per day; meals began to take place in the resident's room, and family members were prevented from meeting the residents) failed in COVID-19 prevention.

The results strongly suggest that not using a mask outside the room significantly predicts SARS-CoV-2 infection in residents (OR: 3.37, 1.74–6.53, *p*. = 0.001), who use a cloth mask, or who do not wear a mask (OR: 2.47, 1.13–5.42, *p* = 0.024) [34]. Masks used properly by staff inside the COVID unit are associated with a lower prevalence of COVID-19 in LTCF [35]. Another vital predictor for residents becoming infected by SARS-CoV-2 is not having a glass barrier in visitors' space (OR: 1.95, 1.11–3.50, *p* = 0.25) [34].

The difficulty in transferring COVID-19 patients to a hospital or other facility (OR = 4.67, p 0.001) was also associated with COVID-19 infection [43].

Active surveillance for guests and the presence of written operational procedures [38], residents' symptom screening [35], the flu vaccine, using masks inside the room, the mask-wearing method, mask change time (daily), and physical distance from a roommate [34] have no significant effect on COVID-19 prevention. Also, conformity to quality standards, compliance, and regular checks of the quality of the cleaning/sanitation/disinfection, availability of hand hygiene supplies and hand hygiene, use of personal protective equipment, and procedure for the management of residents with suspected communicable diseases had no association with the median cumulative incidence of COVID-19 cases among residents [44].

### Combined measures

According to Vijh et al. [41], the combination of four different strategies – case and contact management, proactive case identification, strict infection control procedures, and resource prioritization and stewardship – positively impacts the prevention of COVID-19 transmission.

## Discussion

LTCF remains a high-risk transmission setting where residents and staff are at risk of COVID-19 [47]. Our sample included studies from seven countries, each one under specific government regulations and specific staff qualification levels. This circumstance should be addressed in the analysis of these rsults since it can strongly influence the measures taken locally and the pervasion of COVID in the community where the LTCF is located. However, this information was not always available.

The thirteen included studies identified measures that suggest influencing the outbreak management process in LTCFs. Operational, tactical, and strategic approaches positively prevented the spread of COVID-19 in LTCFs experiencing outbreaks.

We were unable to conduct a meta-analysis due to the heterogeneity between measures, what conditions the correlation between the outcomes and the measures adopted and a meta-analysis [48].

**Fig. 2:**
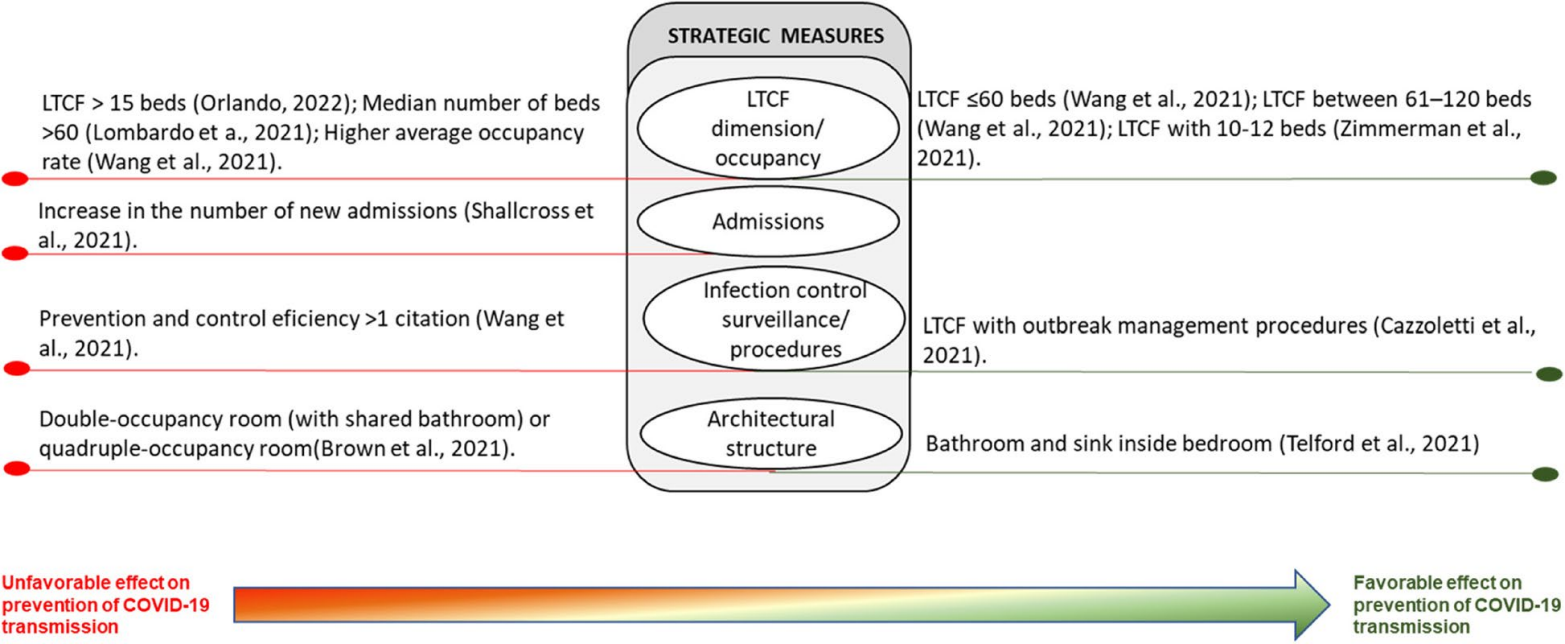
Strategic measures to prevent the spread of COVID-19 in LTCFs experiencing outbreaks

The strategic measures reveal the importance of COVID-19 prevention and control as LTCF structural characteristics, namely the LTCF size, new admissions, infection control surveillance, and architectural structure (Fig. 2). [36, 38, 43]. These findings align with those who claim that single-site institutions have a higher attack rate than sites with multiple units, suggesting that aged care facilities should be designed to be smaller with enough space for social distancing [49].

Vijh's study [50] also concludes that older LTCFs in Canada were associated with the severity of COVID-19 outbreaks. These findings reinforce the importance of regularly assessing infection prevention and control measures tailored to architectural structures and outbreak preparedness in preventing large outbreaks. An integrated surveillance system for influenza, COVID-19, and potentially other respiratory virus infections in LTCF, is urgently needed to develop and sustain resilient responses [51–53].

**Fig. 3:**
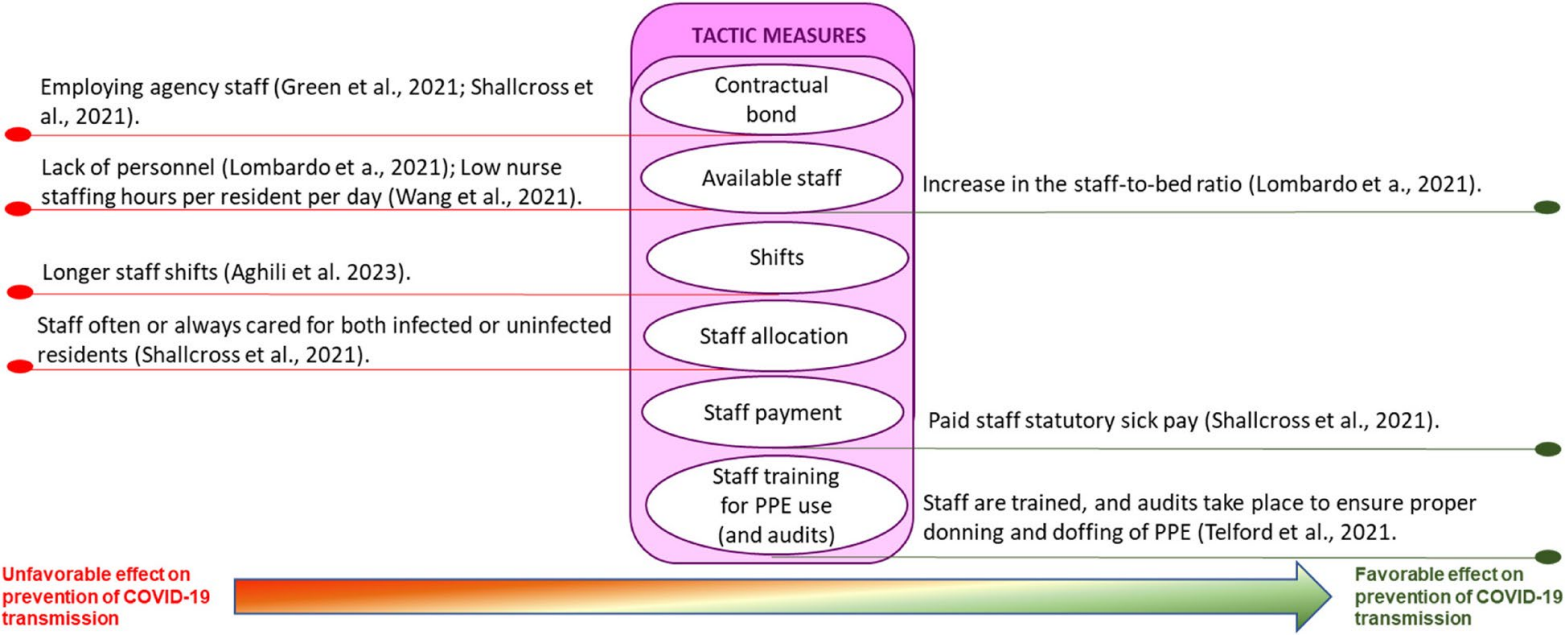
Tactic measures to prevent the spread of COVID-19 in LTCFs experiencing outbreaks

Concerning tactical measures (Fig. 3), the healthcare workers’ conditions at workplaces, mainly nurses working in LTCF, have been paramount in healthcare premises related to infection control, namely, to prevent the spread of the virus, improve care, and reduce the health impact of COVID-19 [54–56]. Other studies suggest that the importation of SARS-CoV-2 by staff from the community is the primary driver of outbreaks [57].

 [34, 36] Our findings support the notion that training, and audits predict lower COVID-19 infection LTCFs [35]. Staff education is beneficial in achieving high adherence to the proper use of PPE and preventing COVID-19 infections in healthcare settings [58].

The LTCF, as units devoted to patients requiring regular supervision and nursing care, are recognised as a good setting for epidemics [59]. The COVID-19 outbreak demanded additional practices besides legislation and policies protecting nurses and patients in LTCF. The recommendations prioritized personal protective equipment and social measures to protect nurses and patients. The health workforce was critical and scarce during the pandemic; however, it is essential to highlight some factors related to working conditions that may influence virus transmission, according to the studies reviewed.

Appropriate measures in LTCF include minimizing exposure, managing absences, and having the correct number of nurses working the proper number of hours in specific sectors (infected and noninfected) [16, 17, 52]. Evidence imposes a necessary concern on the health community, given that it contrasts with the policies adopted in managing nurses to minimize their shortage, such as extending shift hours and hiring work agencies.

Although our study revealed no effect, other studies show that testing prevents the occurrence of an outbreak [42, 60–62]. Outbreak testing should be implemented once it prevents 54% (weekly testing with 48-h test turnaround) to 92% (daily testing with immediate results and 50% relative sensitivity) of SARS-CoV-2 infections [62]. Adding non-outbreak testing could prevent up to an additional 8% of SARS-CoV-2 infections, depending on test frequency and turnaround time [62]. Tsoungui et al. [61] concluded that testing every five days with a good quality test and a processing time of 24 h can lead to a 40% reduction in infections in LTCF.

**Fig. 4:**
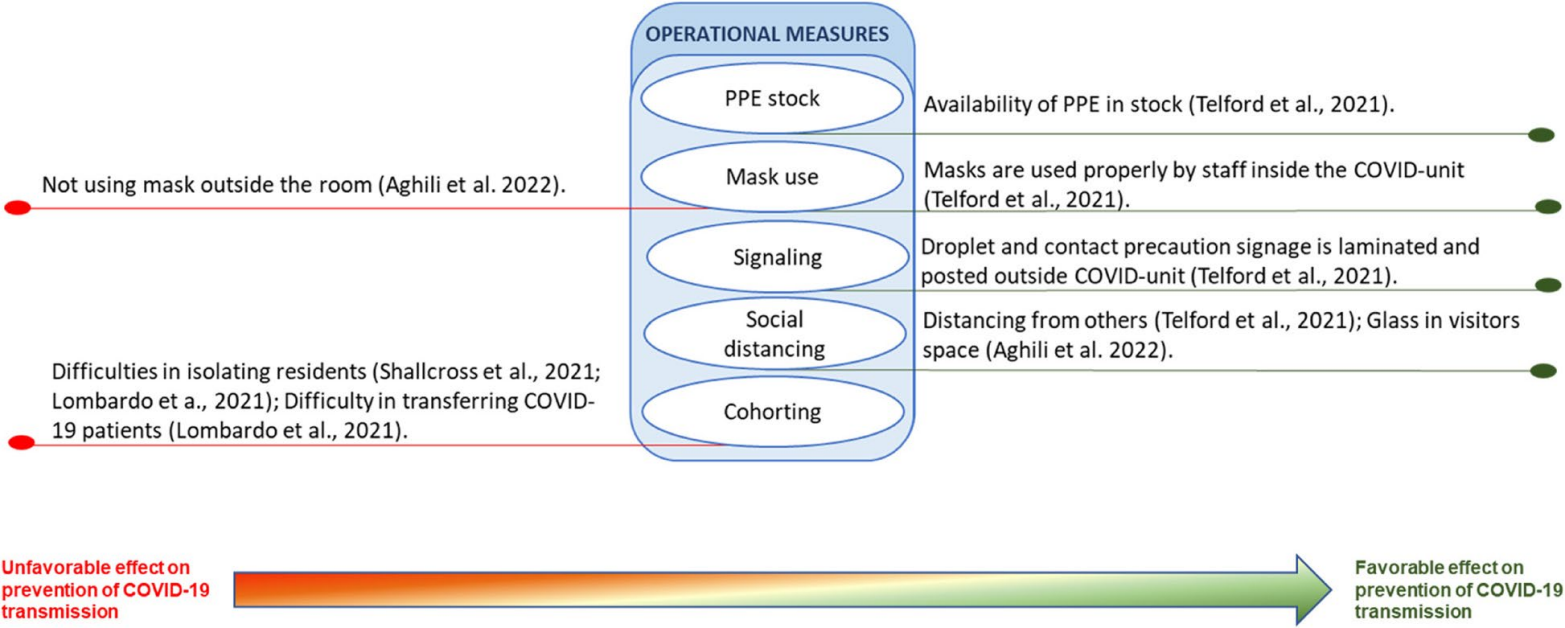
Operational measures to prevent the spread of COVID-19 in LTCFs experiencing outbreaks

The COVID-19 pandemic also underlined the need for special approaches to LTCF at the operational level: personal protective equipment stock, mask use, signaling, social distancing and cohorting (Fig. 4).

Our study confirms that the most important aspect of infection prevention and control is understanding the transmission chain since it permits local and global action on each vector. Schmidt et al. [60] also concluded that applying non-pharmaceutical interventions with increasing rigor reduces the peak of infections. Based on the latest preliminary COVID-19 findings, the WHO [13] released detailed recommendations for using face masks and other personal protective equipment. These safety measures, evaluated during the pandemic, were shown to prevent viral transmission [35], although not using masks has had negative effects [34]. These results are consistent with Bazant´s Guideline about Indoor Airborne Transmission of COVID-19 [63], which recommends the use of the mask. In their work, indoor airborne transmission of COVID-19 depends on ventilation and air filtration, room dimensions, breathing rate, respiratory activity, face mask use of its occupants, and the infectiousness of the respiratory aerosols [43, 44, 46]. The prevention of COVID-19 transmission depends on educating staff, residents, and visitors on infection control and preventive practices [13]. The measures implemented in LTCF brought several negative consequences for residents, staff, and families. Residents' behavioral problems, depression, anxiety, and loneliness were exacerbated by the pandemic and infection control measures [64]. These negative consequences were more likely to affect residents who did not have cognitive impairments [65]. Visitor restrictions greatly impact older adults' and their families' health and well-being [66, 67]. Staff faced several challenges that affected their well-being, like the care of the dying, their suffering, and the ethical, cultural, and spiritual care [68, 69]. Additionally, some evidence [70] reveals that staff in LTCFs had less training, higher staff mobility between working sites, similar personal protective equipment uses, and better self-reported compliance with at-work physical distancing.

The observational design of the bibliographic sample had weak robustness, raising doubts about the generalization of the results. Many infection and control measures integrate complex interventions and are applied in a bundle. In the absence of confounding variable control, it is prudent that these results are seen as suggestive.

**Fig. 5:**
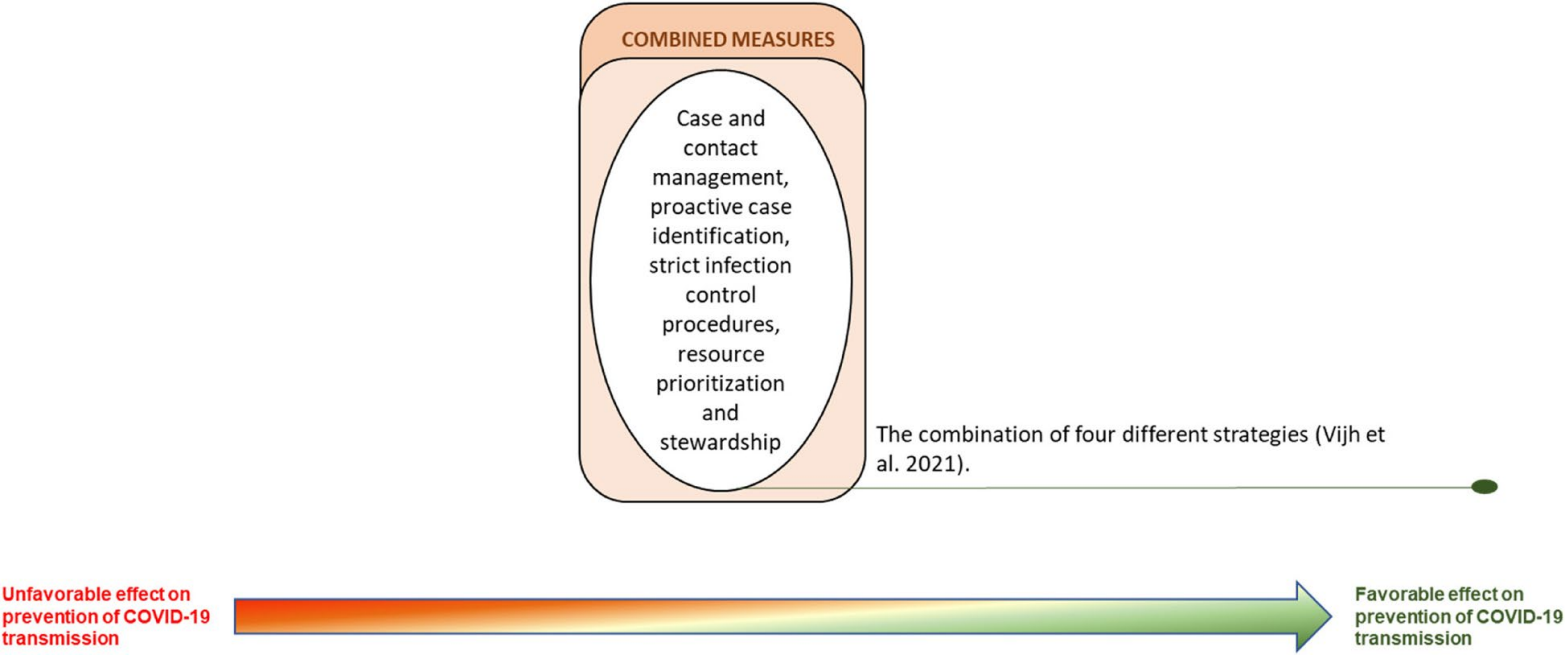
Combined measures to prevent the spread of COVID-19 in LTCFs experiencing outbreaks

The recommendation for applying a multisectoral intervention of combined measures comes from a single study [41] and integrates case and contact management, proactive case identification, strict infection control procedures, resource prioritization and stewardship (Fig. 5).

The application of combined measures is consistent with most available guidance that focus on a set of interventions [51, 55, 71, 72]. However, further research on this matter is needed, with more robust study designs, to elucidate better the implications of the measures in reducing the risk of transmission of covid-19 in LTCF during outbreaks, avoiding measures that might spread COVID-19 and threaten residents, staff, and relatives.

**Fig. 6:**
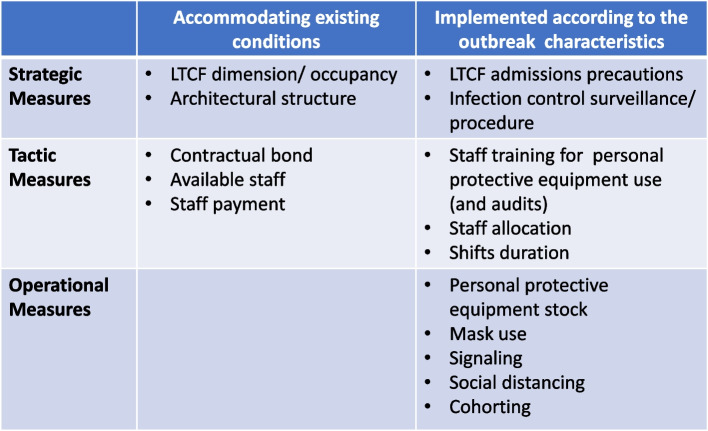
Specific measures for COVID-19 outbreak containment

In each category (strategic, tactic, operational), we can find measures for outbreak containment of two natures: those that are taken in each situation (which result from an adjustment of practices) and those that result from accommodating existing conditions (which point to measures whose implementation are structural and require unique resources, namely financial) (Fig. 6).

Our results show that outbreak control measures, such as admissions precautions, infection control surveillance, staff training and audits, mask use, signalling, and social distancing, overlap with measures to prevent virus entry within the LTCF [15–20]. This evidence highlights the importance of maintaining an ongoing risk assessment and adjusting infection prevention measures in LTCF.

Another noteworthy aspect of our study is that the sample of reports included evaluates interventions, which are consistently implemented in combination. This evidence highlights the importance of investigating outbreak containment measures in LTCF as complex interventions. Previous revisions on infection and control measures for LTCF suggested that combining several infection-control strategies may lower COVID-19 infection and mortality rates [16, 17].

### Strengths and limitations

To the best of our knowledge, this is the first systematic review investigating the effect of containment interventions during COVID-19 outbreaks in LTCF, considering the new coronavirus variants, such as Omicron. The strengths of our review are the comprehensive literature searches and the rigorous methodology. Also, the high rigour level of the design and implementation of this systematic review, including the option for not to include case reports or cross-sectional studies due to their limitations, are strengths that must be highlighted.

The relevance of this study is justified by the continuity of outbreaks in LTCFs and the rapid evolution of studies and publications on the topic, being essential for the development of robust experimental studies that allow the elaboration of supported guidelines of measures to minimize the risk of transmission of COVID-19 in LTCFs and maybe other outbreaks.

However, this review has some limitations. The low number of studies and the impossibility of performing a Meta-analysis due to the methodological and clinical heterogeneity of the studies. Most studies do not consider variables such as country-specific government regulations, the epidemiological context in which the LTCF is inserted, staff qualification levels, and the characteristics that increase vulnerability to residents, such as comorbidities or situations of dependence. The confounding variables are not controlled, which makes it worthwhile to consider the direction of the effect on the spread of the COVID-19 infection during outbreaks in LTCF only suggestive.

## Conclusions

LTCF remains a high-risk transmission setting where residents and staff are at risk of COVID-19. Operational, tactical, and strategic approaches may have a favorable effect on preventing the spread of COVID-19 in LTCFs experiencing outbreaks. Some of these infection prevention and control measures seem to be suitable for preventing COVID-19 dissemination in LTCF. Of course, in designing and using these measures, it is necessary to pay attention to aging people and staff's needs and well-being, as well as work conditions.

Our review showed that there are measures for specific COVID-19 outbreak containment in LTCF that could guide policymakers. The bias assessment of the reviewed articles illuminated constraints in specific papers, underscoring the importance of carefully interpreting the systematic review's conclusions. Acknowledging these limitations is vital for accurately measuring the applicability and reliability of the review's findings, thus ensuring evidence-based decision-making in clinical and research contexts. This systematic review proves the need for higher-quality studies in this domain.Once the COVID-19 pandemic is controlled, and considering that the WHO has declared an end to the public health emergency [73], future studies should focus on endemic situations with similar outbreaks. Similar infection and control interventions should be tested n LTCF to allow comparison across studies and pooling of results to provide robust evidence.

### Supplementary Information

**Additional file 1:** **Supplementary material.** Learning from the covid-19 outbreaks in long-term care facilities: a systematic review.

## Data Availability

All data generated or analyzed during this study are included in this article. Additional information may be requested from the corresponding author.
