# Supplementary material for: Learning from the covid-19 outbreaks in long-term care facilities: a systematic review
Source: BMC Geriatr. 2023 Oct 2;23:618. doi: 10.1186/s12877-023-04319-w (PMC10546730; doi:10.1186/s12877-023-04319-w)
Supplement: Supplementary file 1 — Additional file 1: Supplementary material. Learning from the covid-19 outbreaks in long-term care facilities: a systematic review. [file 12877_2023_4319_MOESM1_ESM.docx]

**SUPPLEMENTARY MATERIAL - LEARNING FROM THE COVID-19 OUTBREAKS IN LONG-TERM CARE FACILITIES: A SYSTEMATIC REVIEW**

| **Case-Control Studies** |  |  |  |  |  |  |  |  |  |  |  |
| --- | --- | --- | --- | --- | --- | --- | --- | --- | --- | --- | --- |
| Checklist | 1 | 2 | 3 | 4 | 5 | 6 | 7 | 8 | 9 | 10 |  |
| Aghili et al. 2022 | Y | Y | Y | Y | Y | Y | U | Y | Y | Y |  |
| Telford et al., 2021 | Y | Y | Y | Y | Y | Y | N | Y | Y | Y |  |
| Wang et al., 2021 | Y | Y | Y | Y | Y | Y | N | Y | Y | Y |  |
| Ohta et al., 2021 | Y | Y | Y | Y | Y | Y | Y | Y | Y | Y |  |
| Orlando 2022 | Y | Y | Y | Y | Y | Y | N | Y | Y | Y |  |
| % | 100 | 100 | 100 | 100 | 100 | 100 | 20 | 100 | 100 | 100 |  |
|  |  |  |  |  |  |  |  |  |  |  |  |
| **Cohort Study** |  |  |  |  |  |  |  |  |  |  |  |
| Checklist | 1 | 2 | 3 | 4 | 5 | 6 | 7 | 8 | 9 | 10 | 11 |
| Brown et al., 2021 | Y | Y | Y | Y | Y | Y | Y | Y | Y | NA | Y |
| Cazzoletti et al., 2021 | Y | Y | Y | Y | Y | Y | Y | Y | Y | NA | Y |
| Green et al., 2021 | Y | Y | Y | N | N | Y | N | Y | Y | NA | Y |
| Lombardo et a., 2021 | Y | Y | Y | Y | Y | Y | N | Y | Y | NA | Y |
| Shallcross et al., 2021 | Y | Y | Y | Y | Y | Y | Y | Y | Y | NA | Y |
| Zimmerman et al., 2021 | Y | U | U | Y | U | Y | Y | Y | Y | NA | Y |
| % | 100 | 83 | 83 | 83 | 67 | 100 | 67 | 100 | 100 | NA | 100 |
|  |  |  |  |  |  |  |  |  |  |  |  |
| **Quasi-Experimental Studies** |  |  |  |  |  |  |  |  |  |  |  |
| Checklist | 1 | 2 | 3 | 4 | 5 | 6 | 7 | 8 | 9 |  |  |
| Stemler et al., 2022 | Y | Y | U | Y | Y | U | Y | Y | Y |  |  |
| Vijh et al., 2021 | Y | Y | Y | Y | Y | Y | Y | Y | Y |  |  |
| **%** | 100 | 100 | 50 | 100 | 100 | 50 | 100 | 100 | 100 |  |  |

Note: Y= Yes, N=Not, U= Unclear, NA= Not applicable; Adapted from: Aromataris E, Munn Z (Editors). JBI Manual for Evidence Synthesis. JBI, 2020. Available from <https://synthesismanual.jbi.global>.
